# Rapid Assessment of Avoidable Blindness in the Occupied Palestinian Territories

**DOI:** 10.1371/journal.pone.0011854

**Published:** 2010-07-29

**Authors:** Far Chiang, Hannah Kuper, Robert Lindfield, Tiarnan Keenan, Na'el Seyam, Denise Magauran, Nasrallah Khalilia, Habes Batta, Ziad Abdeen, Nicholas Sargent

**Affiliations:** 1 St. John Eye Hospital, Jerusalem, Occupied Palestinian Territories; 2 London School of Hygiene and Tropical Medicine, London, United Kingdom; 3 Manchester Royal Eye Hospital, Manchester, United Kingdom; 4 Military Medical Services, Gaza, Occupied Palestinian Territories; 5 Epsom and St. Helier University Hospitals NHS Trust, Sutton, United Kingdom; 6 Al-Quds Nutrition and Health Research Institute, Al Quds University, Jerusalem, Occupied Palestinian Territories; Helmholtz Zentrum München, Germany

## Abstract

**Background:**

There are no recent data on the prevalence and causes of blindness in the Occupied Palestinian Territories. The aim of our study was to estimate the prevalence and causes of blindness and visual impairment in the population aged 50 years and above in the Occupied Palestinian Territories using the Rapid Assessment of Avoidable Blindness (RAAB) survey method.

**Methods and Findings:**

Clusters of 40 people who were 50 years and above were selected with probability proportionate to size using a multistage cluster random sampling method. Participants received a comprehensive ophthalmic examination in their homes, including visual acuity testing by one of three experienced ophthalmologists. The principal cause for visual loss was determined by an experienced ophthalmologist using portable diagnostic instruments. Information about previous cataract surgery, satisfaction with surgery and barriers to cataract surgery were collected. The prevalence of self-reported diabetes was also determined. The prevalence of bilateral blindness (VA<3/60 in the better eye with available correction) was 3.4% (95% CI: 2.7–4.0), 2.0% (95% CI: 1.4–2.5) for severe visual impairment (VA≥3/60 and <6/60), and 7.4% (95% CI: 6.4–8.3) for visual impairment (VA≥6/60 and <6/18). Avoidable causes (i.e. cataract, refractive error, aphakia, surgical complications, corneal scarring and phthysis) accounted for 80.0% of bilateral blindness, severe visual impairment (70.7%) and visual impairment (86.2%). Cataract was the main cause of blindness (55.0%). The prevalence of blindness was higher in Gaza (4.9%, 95% CI: 3.7–6.1%) than in the West Bank (2.5%, 95% CI: 1.9–3.1%) and among women (4.3%,95% CI: 3.3–5.2%) compared to men (2.2%,95%CI:1.5–2.9%). Among people who had undergone cataract surgery in the past, only 54.5% of eyes obtained a good outcome (VA≥6/18), 23.2% had a borderline outcome (VA<6/18 and ≥6/60) and 22.3% had a poor outcome (VA<6/60) with available correction. The prevalence of self-reported diabetes mellitus in ≥50 year age group was 26.4% (95% CI: 24.9–27.9).

**Conclusions:**

The prevalence of blindness suggests that significant numbers of people in the Occupied Palestinian Territories exist who do not access eye care - predominantly women and those residing in Gaza. Programmes need to focus on maximizing the use of current services by these excluded groups.

## Introduction

The World Health Organisation (WHO) estimates that there are 45 million people in the world who are blind (vision worse than 3/60 in the better eye with presenting vision). This is expected to rise to 76 million by 2020 if current services are not improved. VISION 2020 is a joint initiative by the WHO and the International Association for the Prevention of Blindness that aims to eliminate avoidable blindness by the year 2020. The first step in achieving this target is to obtain baseline data on visual impairment at country and district levels for planning and monitoring eye care programmes.

The prevalence of blindness differs between countries [1], and may be increased four-fold in areas affected by violent conflict [2]. The WHO estimates that the number of blind people in 2002 in the Eastern Mediterranean Region-B (which includes the Occupied Palestinian Territories) was over 1 million people, and that the prevalence of blindness in adults aged 50 years and above in this region was 5.6% [1].

The Occupied Palestinian Territories comprise East Jerusalem, the West Bank and the Gaza Strip. The total population of the Occupied Palestinian Territories in 2007 (figures available at the time of the study) was estimated at 3,761,600, with 2,345,100 living in East Jerusalem and the West Bank, and 1,416,500 in the Gaza Strip [3]–[4]. Military occupation means that people with an identity card recognising them as Palestinians from the West Bank or Gaza can enter Jerusalem via military checkpoints only with a special permit. Such permits are not always granted thus restricting access to secondary and tertiary referral centres in East Jerusalem [5]–[8].

No robust or comprehensive study of blindness in Palestinians has been conducted for over 20 years. The most recent population-based study of blindness in the OPT was conducted in 1984 and included 9,054 people selected by cluster random sampling [9]. The estimated prevalence of blindness for all ages (including refractive error) was 1.7% and for visual impairment was 6.8%. The major causes of blindness were cataract (31.6%) and trachoma (13.4%). However, substantial demographic changes have occurred in the Occupied Palestinian Territories over the past 20 years, including a large increase in the Palestinian population. A study conducted in 1990 by the visiting Russian ophthalmologist, Golychev, reviewed people presenting at clinics set up in both the West Bank and Gaza [10]. The main cause of blindness was found to be corneal opacification of all types. As this was a sample of convenience, selection bias prevents the results from being assumed to be reflective of the population. Surveys conducted in nearby countries have produced prevalence estimates for blindness from 0.6% and 0.7% in Lebanon and Saudi Arabia, to 3.2% in Pakistan and Oman [1], [11]–[12].

Current ophthalmic services for Palestinians include clinics and hospitals run by the Palestinian Authority hospitals, non-governmental organisations (NGOs) and the private sector. The St John Eye Hospital network is a NGO that includes the main hospital in East Jerusalem, permanent clinics in Gaza, Hebron and Anabta, and two mobile outreach services operating in the West Bank. Established in 1882 as a charitable organisation, St John Eye Hospital is the largest single provider of ophthalmic care in the Occupied Palestinian Territories, which it provides to all people irrespective of race, religion or ability to pay. The Palestinian Authority delivers ophthalmic services in the West Bank through Rafidia Hospital (located in Nablus), and in the Gaza Strip through Nasser Hospital and European Gaza Hospital. There is financial cover for treatment of members in the Palestinian Authority national medical insurance scheme in non-governmental centres [8]. Other NGO service providers include the Bethlehem Arab Society for Rehabilitation (located in Beit Jala), which conducts intraocular surgery for the Bethlehem area. Finally, many private clinics operate in the West Bank and Gaza Strip. The Palestinian Society of Ophthalmologists has 68 licensed ophthalmologists in the West Bank, and 74 in the Gaza Strip, though the majority of these do not perform cataract surgery largely due to a lack of training. Those needing services also seek eye care in Israel and neighbouring Arab countries including Egypt and Jordan.

The aim of this study was to conduct a Rapid Assessment of Avoidable Blindness (RAAB) in people aged 50 years and above in the Occupied Palestinian Territories, in order to estimate the magnitude and causes of blindness and visual impairment, and to provide information that would enhance the impact of services and resources for this area.

## Methods

### Sample Size Calculation

At the time of the survey, approximately 9% (338,400) of the total Palestinian population in the Occupied Palestinian Territories (3,761,600) was over 50 years old [3], [13]. To estimate the required sample size we assumed the following: expected prevalence of blindness 4%, desired precision 20% (worst acceptable result 4.8%), confidence level 95%, and non-response rate 15%. In addition, the required sample size was increased because of the use of cluster rather than simple random sampling, and we used a design effect of 1.4 for this purpose. The sample size required calculated using the RAAB software (http://www.cehjournal.org/files/s0701.html) was 3,800 individuals, corresponding to 95 clusters of 40 people.

### Sampling Frame

Data from the Palestinian Central Bureau of Statistics 1997 were used as the sampling frame, updated with 2007 growth rates (by governorate) to generate up-to-date population estimates for each enumeration area [13]–[15]. From the enumeration areas, 95 clusters were selected using probability proportionate to size, including 3 clusters located in East Jerusalem from neighbourhoods included in the Palestinian Authority census. In the conflict area of Beit Hanoun, Gaza, 3 clusters were replaced with locations in the West Bank (due to the logistical constraints of working in Gaza it was not possible to replace these with clusters from Gaza).

The examination team sequentially visited households until 40 people ≥50 years were identified. If an eligible person was absent, the examination team returned twice to the household on the same day to examine the individual before leaving the area. For eligible individuals who could not be examined on the day, information on visual status was collected from relatives or neighbours. Non-responders were not included in the analyses nor in the estimates of prevalence of blindness, but information was collected on their visual status to allow assessment of whether non-attendance introduced bias in the estimate of blindness. If 40 people were not found in the segment, then sampling was continued in the most adjacent segment until the target number of 40 people was reached. The survey was carried out over an eight-week period (June-July in the West Bank, late June to mid-August in the Gaza Strip) in 2008.

### Ophthalmic evaluation and data collection

The standardised set of questions was completed for each eligible person. They were divided into seven parts including: general demographic information; whether known to have diabetes mellitus; visual acuity and lens assessment; principal cause of visual impairment; reasons why cataract surgery had not been done; and information about cataract surgery done including time, place and level of patient satisfaction.

VA was measured using a Snellen tumbling ‘E’ chart, which had an optotype size 6/18 on one side and 6/60 on the other side at a 6 or 3 metre distance, and was measured in full daylight with available correction. Pinhole VA was measured when the VA was <6/18 in either eye. The lens was examined in both eyes using a direct ophthalmoscope or portable slit-lamp and was graded into the following categories; ‘normal lens’, ‘obvious lens opacity’, ‘lens absent (aphakia)’ or ‘IOL implantation’. If corneal opacification precluded lens examination then ‘no view of lens’ was recorded.

If the VA improved to 6/18 or better with pin-hole, then refractive error was assigned as the cause of visual impairment. If it was found that the vision in either eye was <6/18 with a pinhole, a more detailed examination was performed of the anterior segment to elicit the cause. If the cause was not in the anterior segment, the ophthalmologist proceeded to perform fundoscopy with a mydriatic eye drop. Glaucoma was defined as the principal cause of visual impairment if the optic cup-to-disc ratio was greater than 0.6, in the absence of another cause of reduced vision.

The principal cause of visual impairment was based on the WHO convention whereby the principal cause is attributed to the primary disorder. If there are multiple causes, then following this convention, the cause that can be most easily treated was assigned as the principal cause [16].

### Categories of visual impairment

This study used the WHO categories for visual impairment. Based on presenting visual acuity (VA) with available correction in the better eye, the three categories are defined as follows: Blindness, VA<3/60 in the better eye; severe visual impairment, VA<6/60 but≥3/60 in the better eye; visual impairment, VA<6/18 but ≥6/60 [19].

### Training and intervariability testing of survey teams

The RAAB methodology required three teams, each comprising an experienced ophthalmologist, an ophthalmic assistant and a driver. Two teams were assigned to the West Bank, and one to the Gaza Strip. All teams received one week of training in both theoretical and practical aspects of the RAAB methodology by certified RAAB trainers. Inter-observer variation between all teams was measured using repeat examination of 40 participants. The assessment of VA, lens examination and assigned cause of blindness were compared to ensure a high degree of consistency between teams. The kappa statistic was >0.60 for all these measures and so the teams were deemed to be consistent in their assessments. The training programme also included a pilot visit to a field location with the trainers.

### Statistical Analysis

The survey software program ‘RAAB version 4.02’ (Health Information Services and Tax Software) was used. This software allows data entry, sample size calculation and inter-observer variability testing as well as standardised data analysis. The prevalence estimates took account of the design effect when estimating the confidence intervals because cluster sample was used rather than simple random sampling.. The cataract surgical coverage of people was defined as the proportion of people needing surgery who had undergone cataract surgery. This is calculated as:

Cataract Surgical Coverage
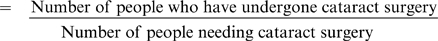


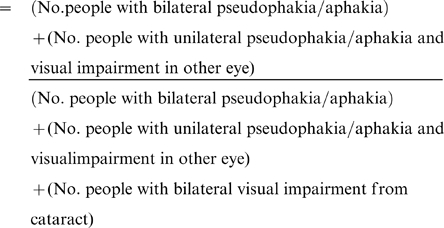



For these purposes visual impairment was defined using pinhole vision. Cataract surgical coverage was also calculated for eyes. Since the VA prior to surgery was not known, we assumed, in turn, that only patients with VA below a certain threshold (<3/60, <6/60, <6/18 respectively) received surgery for their cataract. Estimates of the number of cases with blindness, severe visual impairment and visual impairment were obtained by extrapolating the age-sex-specific prevalence estimates to the age-sex structure of the population and these were rounded to the nearest 10.

### Ethical approval

The study was approved and endorsed by the Palestinian Ministry of Health. Ethics approval was granted by the St John Eye Hospital Ethics Committee and the research followed the tenets of the Declaration of Helsinki. Informed verbal consent was obtained for each person included in the study. This method of consent was specifically approved by the same ethics committee in light of a two day pilot showing many Palestinians refuse to sign any paperwork to unknown visitors. Claims exist that Jewish settlement groups have tricked Palestinians into signing away property rights under false pretences. An explanation of the nature and the aims of the nature and possible consequences of the study were provided to participants. All participants received feedback about their eyes and were advised to seek ophthalmic attention if they had any concerns. People who were discovered to have eye problems were given relevant counseling and advice. Those whom it was thought would benefit from further management, including those with operable cataracts, were referred to a St. John facility for a free consultation unless they were already receiving treatment from an ophthalmologist.

## Results

Of the 3,800 selected participants aged ≥50 years, 3,579 were examined (response rate of 94.2%). Of the 221 non-respondents, 177 (4.7%) were not available, 29 (8%) refused to participate and 15 (0.4%) were not capable of being examined. Those examined were representative of the population of the Occupied Palestinian Territories in terms of age and sex distribution (Table 1). The majority who were not available were men (72.3%). More women refused participation (62.1%) compared to men (37.9%). Among the non-responders, 9 were reported to be believed to be blind (4%).

**Table 1:** Composition of population in sample and in survey area, by age and sex for the Occupied Palestinian Territories.

	Occupied Palestinian Territories		West Bank		Gaza	
	Survey area (N)*	Examined (n)	Survey area(N)*	Examined(n)	Survey area(N)*	Examined(n)
Age group						
50–59 y	141,900 (42.5%)	1,625 (45.4%)	94,500 (42.1%)	1,052 (45.9%)	47,400 (43.6%)	573 (44.4%)
60–69 y	110,600 (33.2%)	1,002 (28.0%)	73,500 (32.8%)	612 (26.7%)	37,100 (34.1%)	390 (30.2%)
70–79 y	57,000 (17.1%)	674 (18.8%)	39,500 (17.6%)	432 (18.9%)	17,500 (16.1%)	242 (18.8%)
80+ y	23,900 (7.2%)	278 (7.8%)	17,000 (7.6%)	194 (8.5%)	6,900 (6.3%)	84 (6.5%)
Men	148300 (44.5%)	1,618 (45.2%)	100,400 (44.8%)	1,036 (45.2%)	47,800 (43.9%)	582 (45.1%)
Women	185100 (55.5%)	1,961 (54.8%)	124,000 (55.2%)	1,254 (54.8%)	61,100 (56.1%)	707 (54.8%)
Total	333,400	3,579	224,000	2,290	109,000	1,289

*Source: Palestinian Central Bureau of Statistics [4], [16].

The prevalence of blindness, based on presenting vision, in the ≥50 years age group was 3.4% (95% CI: 2.7–4.0%). The prevalence was 2.6% (95% CI: 2.0–3.1%) for severe visual impairment and 10.7% (95% CI: 9.4–12.0%) for visual impairment (Table 2). Bilateral blindness was twice as prevalent in Gaza (4.9%, 95% CI: 3.7–6.1%) as in the West Bank (2.5%, 95% CI: 1.9–3.1%). In addition, both severe visual impairment and visual impairment were more prevalent in Gaza (3.2% and 12.5%) than the West Bank (2.2% and 9.7%). Unilateral blindness was more than twice as common as binocular blindness (8.2%, 95% CI: 7.3–9.1%). Compared to men women had a higher prevalence of blindness, severe visual impairment and visual impairment, but this only reached statistical significance for blindness (p = 0.0007) and not for severe visual impairment (p = 0.58) or visual impairment (p = 0.50). The prevalence of self-reported diabetes mellitus in ≥50 years age group was 26.4% (95% CI: 24.9–27.9%).

**Table 2:** Prevalence of Blindness, Severe Visual Impairment, and Visual Impairment in the Occupied Palestinian Territories (with available correction in the better eye in adults aged 50 years and above).

Area	Sex	Total number	Bilateral blindness (VA<3/60)		Bilateral severe visual impairment (VA<6/60 and VA≥3/60)		Bilateral visual impairment (VA<6/18 and VA≥6/60)	
			Number	Prevalence (95% CI)	Number	Prevalence (95% CI)	Number	Prevalence (95% CI)
Occupied Palestinian Territories	Men	1618	36	2.2% (1.5–2.9%)	39	2.4% (1.6–3.2%)	167	10.3% (8.7–12.0%)
	Women	1961	84	4.3% (3.3–5.2%)	53	2.7% (1.9–3.5%	216	11.0% (9.5–12.6%)
	Total	3579	120	3.4% (2.7–4.0%)	92	2.6% (2.0–3.1%)	383	10.7% (9.4–12.0%)
West Bank	Men	1036	19	1.8% (1.0–2.7%)	24	2.3% (1.4–3.3%)	104	10.0% (7.8–12.3%)
	Women	1254	38	3.0% (2.1–4.0%)	27	2.1% (1.4–3.0%)	118	9.4% (7.7–11.1%)
	Total	2,290	57	2.5% (1.9–3.1%)	51	2.2% (1.6–2.9%)	222	9.7% (8.1–11.3%)
Gaza	Men	582	17	2.9% (1.6–4.2%)	15	2.6% (1.3–3.9%)	63	10.8% (8.6–13.1%)
	Women	707	46	6.5% (4.6–8.4%)	26	3.7% (2.2–5.2%)	98	13.9% (11.0–16.8%)
	Total	1,289	63	4.9% (3.7–6.1%)	41	3.2% (2.1–4.2%)	161	12.5% (10.4–14.6%)

Avoidable causes of blindness (cataract, refractive error, corneal scar, surgical complications, trachoma and phthysis) accounted for 80.0% of the total amount of blindness, 70.7% of severe visual impairment and 86.2% of visual impairment in the Occupied Palestinian Territories (Table 3). Cataract was the dominant cause of blindness (55.0%), severe visual impairment (56.5%), and visual impairment (45.2%). Corneal scarring was also an important cause of blindness (14.2%) and severe visual impairment (7.6%). Diabetic retinopathy accounted for 8.3% of blindness, 14.1% of severe visual loss and 5.5% of visual impairment. Glaucoma was responsible for 5.8% of blindness. Refractive error was an important cause of visual impairment (36.3%), but not severe visual impairment or blindness. The prevalence of blindness from bilateral cataract for the Occupied Palestinian Territories was 1.23% (95%CI 0.86%–1.60%) with the prevalence being higher in Gaza (1.86%, 95%CI 1.11%–2.61%) compared to the West Bank (0.87%, 95%CI 0.49%–1.25%)

**Table 3:** Proportion of Blindness, Severe Visual Impairment, and Visual Impairment due to specific causes in the Occupied Palestinian Territories.

Cause	Blindness (VA<3/60 with available correction)			Severe visual impairment (VA<6/60 to 3/60 with available correction)			Visual impairment (VA<6/18 to 6/60 with available correction)		
	Occupied Palestinian Territories	West Bank	Gaza	Occupied Palestinian Territories	West Bank	Gaza	Occupied Palestinian Territories	West Bank	Gaza
	n = 120	n = 57	n = 63	n = 92	n = 51	n = 41	n = 383	n = 222	n = 161
**Avoidable causes**									
Refractive error	1.7%	1.8%	1.6%	2.2%	0	4.9%	36.3%	37.8%	34.2%
Cataract	55.0%	56.1%	54.0%	56.5%	62.7%	48.8%	45.2%	43.7%	47.2%
Aphakia (uncorrected)	0	0	0	0	0	0	0.3%	0	0.6%
Surgery related	5.0%	3.5%	6.3%	4.3%	2.0%	7.3%	2.3%	1.8%	3.1%
Corneal Scar (non-trachomatous)	14.2%	19.3%	9.5%	7.6%	7.8%	7.3%	1.8%	2.7%	0.6%
Phthysis	2.5%	3.5%	1.6%	0	0	0	0	0	0
Trachoma	1.7%	1.8%	1.6%	0	0	0	0.3%	0	0.6%
Total Avoidable*	80.0%	86.0%	74.6%	70.7%	72.5%	68.3%	86.2%	86.0%	86.3%
**Potentially avoidable and other causes** **									
Diabetic retinopathy	8.3%	7.0%	9.5%	14.1%	11.8%	17.1%	5.5%	4.5%	6.8%
Glaucoma	5.8%	1.8%	9.5%	3.3%	3.9%	2.4%	0.3%	0.5%	0
ARMD	2.5%	3.5%	1.6%	4.3%	7.8%	0	2.3%	2.7%	1.9%
Other posterior segment/CNS	3.3%	1.8%	4.8%	6.5%	2.0%	12.2%	5.5%	5.9%	5.0%
Globe abnormality	0	0	0	1.1%	2.0%	0	0.3%	0.5%	0

*Refractive error, cataract, aphakia (uncorrected), surgical complications, corneal scar, phthysis, trachoma.

**Diabetic retinopathy, glaucoma, age-related macular degeneration (ARMD), other posterior segment and central nervous system diseases, globe abnormalities.

The cataract surgical coverage was high with 86% of people requiring surgery at VA<3/60 level having received surgery (Table 4). This was even higher in men (91.5%) than in women (82.5%). For people with VA<6/60 and VA<6/18, 80.7% and 61.8% of those needing surgery had received it, respectively.

**Table 4:** *Cataract Surgical Coverage (CSC) by person and eyes in the Occupied Palestinian Territories.

	Occupied Palestinian Territories		West Bank		Gaza	
	CSC-Persons	CSC-Eyes	CSC-Persons	CSC-Eyes	CSC-Persons	CSC-Eyes
VA<3/60						
Men	91.5%	69.0%	90.9%	67.7%	91.6%	70.4%
Women	82.5%	59.5%	87.6%	63.0%	77.0%	56.3%
Total	86.0%	63.4%	89.9%	65.0%	82.6%	61.6%
VA<6/60						
Men	86.1%	61.3%	85.3%	60.0%	87.1%	62.9%
Women	77.0%	53.8%	80.1%	56.9%	74.0%	51.0%
Total	80.7%	56.9%	82.2%	58.2%	79.2%	55.5%
VA<6/18						
Men	65.1%	44.9%	64.5%	44.6%	65.4%	44.5%
Women	59.5%	37.7%	65.4%	42.0%	54.1%	34.0%
Total	61.8%	40.6%	65.0%	43.1%	58.5%	37.9%

*with pin-hole vision in the better eye in adults aged 50 years and above.

The vast majority of people who had undergone cataract surgery (94.3%) had received an intraocular lens. However, only 54.5% of eyes obtained a good post-operative outcome (VA≥6/18), 23.2% had a borderline outcome (VA<6/18 and≥6/60) and 22.3% had a poor outcome (VA<6/60) with available correction (Table 5). This fails to meet the WHO target >80% of eyes having good vision with available correction. The proportion of eyes that had a good outcome was slightly higher in those eyes that were operated within 5 years of the survey (58.3%) compared to those eyes operated over 5 years before the survey (50.2%). Outcomes were a little higher in the West Bank than in Gaza (57.5% had good outcome compared to 50.2% respectively). In those eyes with a poor outcome (VA<6/60 with available correction), the single largest cause was attributable to ocular co-morbidity (e.g. ARMD, glaucoma, diabetic retinopathy) in 59.5%, followed by surgical complications in 28.1%. This contrasted with the single largest cause of visual impairment (VA<6/18) in eyes following cataract surgery, which was lack of spectacles (35.7%). Despite the poor results, most of the people who received cataract surgery in the Occupied Palestinian Territories were very satisfied (54%) or partially satisfied (30%).

**Table 5:** Visual acuity in eyes following cataract surgery in the Occupied Palestinian Territories.

Post-operative visual acuity	Occupied Palestinian Territories	West Bank	Gaza	WHO recommended standard
	n = 543	n = 320	N = 223	
Available correction				
Good (VA 6/6–6/18)	296 (54.5%)	184 (57.5%)	112 (50.2%)	>80%
Borderline (VA 6/18–6/60)	126 (23.2%)	70 (21.9%)	56 (25.1%)	<15%
Poor (VA<6/60)	121 (22.3%)	66 (20.6%)	55 (24.7%)	<5%
Best correction				
Good (VA 6/6–6/18)	357 (65.7%)	220 (68.8%)	137 (61.4%)	>90%
Borderline (VA 6/18–6/60)	87 (16.0%)	42 (13.1%)	45 (20.2%)	<5%
Poor (VA<6/60)	99 (18.2%)	58 (18.1%)	41 (18.4%)	<5%

Up to two reasons could be given for not obtaining cataract surgery. The most common reasons cited by people with bilateral vision ≤6/60 due to cataract (were “Old age and need not felt” (33%), “Could not afford” (20%), “Contraindication” (15%), “Fear of operation (14%) and “Unaware of treatment” (6%). Women were more likely than men to cite “Cannot afford” (21% and 17% respectively) and “Fear of operation” (19% and 0% respectively). The problem of obtaining permits to pass through military checkpoints within the West Bank (including East Jerusalem) was cited by 11% as a barrier to obtaining cataract surgery.

### Population estimates

Extrapolating the survey estimates to the age and sex distribution of the Occupied Palestinian Territories from the updated census data suggests that in the ≥50 years age group, there are an estimated 10,870 blind people (95% CI: 8,560–12,850), 8,810 (95% CI: 7,120–10,850) severely visually impaired people and 36,010 (95% CI: 31,670–40,340) visually impaired people (Table 6). The all-age prevalence of blindness for the Occupied Palestinian Territories is estimated to be 0.4%, based on the WHO estimate that 82% of blindness occurs in ≥50 years. This equates to 13,300 people out of a population of 3.7 million.

**Table 6:** Extrapolated estimates for the number of Blind, Severe Visually Impaired and Visually Impaired in the Occupied Palestinian Territories.

VA with available correction	Bilateral blindness		Bilateral severe visual impairment		Bilateral visual impairment	
	Number	(95% CI)	Number	(95% CI)	Number	(95% CI)
Occupied Palestinian Territories						
Men	3,180	(2,120–4,230)	3,570	(2,430–4,710)	15,210	(12,760–17650)
Women	7,700	(5,920–9,470)	5,240	(3,830–6,650)	20,800	(17,910–23,690)
Total	10,870	(8,770–12,970)	8,810	(6,910–10,720)	36,010	(31,770–40,240)
West Bank						
Men	1,740	(900–2,570)	2,330	(1,370–3,300)	10,050	(7,780–12,220)
Women	3,730	(2,590–4,870)	2,790	(1,800–3,790)	11,810	(9,700–13,920)
Total	5,470	(4,080–6,860)	5,130	(3,640–6,610)	21,860	(19,620–24,110)
Gaza						
Men	1,370	(740–2,000)	1,140	(520–1,770)	5,170	(4,100–6,250)
Women	3,540	(2,390–4,690)	2,300	(1,390–3,220)	8,480	(6,720–10,250)
Total	4,910	(3,600–6,220)	3,450	(2,360–4,540)	13,660	(11,340–15,970)

Using the estimates of the prevalence of cataract in the Occupied Palestinian Territories, it is expected that in the ≥50 years group, cataract accounts for 6,000 blind, 5,000 severely visually impaired, and 16,300 visually impaired. To reach the Vision 2020 target of operating on 20% of the prevalence of cataract per year causing VA<6/60 bilaterally; 2,200 cataract operations on separate people would need to be performed per year.

## Discussion

RAAB surveys have been conducted in several countries [18]–[24]. This was the first to be completed in the Middle East, providing an essential baseline for Vision 2020 programmes in the Occupied Palestinian Territories. The prevalence of bilateral blindness (VA<3/60 in the better eye with available correction) was 3.4% (95% CI: 2.7–4.0) in those people aged ≥50 years. This gave rise to an estimate of blindness for the total population of 0.4%, which was half of that previously estimated for the EMR-B (0.8%) [1]. The prevalence of blindness was higher in Gaza (4.9%, 95% CI: 3.7–6.1%) than in the West Bank (2.5%, 95% CI: 1.9–3.1%) and among women (4.3%,95% CI: 3.3–5.2%) compared to men (2.2%,95% CI:1.5–2.9%).

Avoidable causes (i.e. cataract, refractive error, aphakia, surgical complications, corneal scarring and phthysis) accounted for 80.0% of bilateral blindness and cataract was the main cause of blindness (55.0%). This dispels the conclusions of a previous clinic-based study that suggested that corneal scarring was the main cause of blindness in the region [10]. A smaller proportion of the cases of blindness were attributable to trachoma (1.7%) compared with the results of a previous study (13.4%) [9]. Outcome after cataract surgery was found to be inadequate as only half of eyes that had undergone surgery had achieved a good outcome (VA≥6/18).

Unlike the Occupied Palestinian Territories, neighbouring Israel (not included in the Eastern Mediterranean Region, but included in the ‘European Region’ by the WHO) has a much lower estimated prevalence of blindness in the population (0.3%) compared to the Eastern Mediterranean Region (0.8%), and has a profile for causes of blindness similar to relatively wealthy nations such as the UK with age-related macular degeneration and glaucoma being the two main causes of blindness [25]–[26]. In neighbouring Lebanon, a national survey including people who were three years and above gave a slightly higher estimate compared to the Occupied Palestinian Territories for the prevalence of blindness of 0.6% and similarly found cataract as the leading cause of blindness (41%) [11]. Refractive errors, however, were the second largest cause (13%) followed by non-trachomatous corneal opacification (7.5%). Oman, located in the Arabian Peninsula, is much wealthier than the Occupied Palestinian Territories, yet was estimated to have a higher prevalence of blindness in a survey including all age groups of 1.1% [12]. However, the two largest causes of blindness were also cataract (30.5%) and trachomatous corneal scarring (24%).

The results of the survey suggest that approximately 6,000 people in the Occupied Palestinian Territories are blind due to cataract. The reasons given for not attending for cataract surgery suggest education and beliefs play the largest factor in the under-utilisation of services by the blind and that merely increasing the number of cataract surgeons and operations may not in itself effectively reduce the number of blind people in the population. Any expansion of surgical services need to be coupled with education programmes that raise public awareness and support health services to provide early identification and referral.

Those participants with visual impairment due to cataract regarded the process of obtaining permits and getting through military checkpoints (where access can still be refused with or without a permit), as a barrier. The prevalence of blindness in Gaza was twice that of the West Bank, where unemployment, poverty, and access to outside health services (such as Egypt and Jerusalem are worse than in the West Bank.

Our study points to inequity between the sexes with women having a greater prevalence of blindness and lower cataract surgical coverage. This has been found in other RAAB surveys in other countries [19]–[22]. Women in our study were only marginally more likely to express poverty as a reason not to have cataract surgery compared to men. Studies from Egypt have discussed and suggested that such gender disparity may be partly due to the preference of women to seek opinions for eye care no further than other family members and traditional healers [27]–[28]. Further anthropological studies may confirm if a similar pattern occurs in the Palestinian population.

The high proportion of participants with a poor outcome after surgery is of concern and requires further research and auditing of surgical cases to identify possible ways of reducing it. Post-operative refractive error was a major cause of poor and borderline outcome. The provision of glasses will reduce the proportion of visually impaired people after cataract surgery.

The number of people with diabetes mellitus in the Middle East is expected to grow from the 2000 estimate of 20 million to just under 60 million in 2030 [29]. The prevalence of self-reported diabetes in the >50 years age group was high (26.4%, 95% CI: 24.9–27.9), and the real estimate is likely to be higher still when considering the number of undiagnosed cases in the community. Our estimate is comparable to the prevalence of diabetes obtained from routine data of the UN Relief and Works Agency (19.1% at 50–59 years and 24.8% at ≥60 years) [7]. The high proportion of people with <6/60 visual acuity due to diabetic retinopathy points to an urgent need to plan future diabetic eye services.

Our study highlights the problem of conducting population-based studies in areas of conflict and military occupation. The decision to carry out reselection of three clusters in Gaza due to security reasons was not taken lightly but was deemed necessary. Since the prevalence of blindness was higher in Gaza than in the West Bank this could have caused a slight under-estimation of the overall prevalence of blindness. Although East Jerusalem was included in the sampling frame, no clusters in East Jerusalem were selected in the randomised selection process. Estimation of the proportion of people with posterior segment disease and glaucoma may have been imprecise because detailed examination was not always possible. Applanation tonometry was abandoned at the trial stage when it was realised that written consent for the procedure aroused suspicion in a population experiencing problems with land rights.

Confidence in the estimates for our study can be based on several strengths. All teams received organised training and were regularly monitored in the field. Selection bias was likely to be low as we achieved a high response rate and the prevalence of blindness was similar in responders compared to non-responders. Information bias including observer bias was reduced by all teams receiving training using experienced ophthalmologists and by having inter-observer variability tested objectively. Further quality control in the field was ensured by monitoring.

In conclusion, our study shows that despite the lower than predicted level of blindness, most blindness in the Occupied Palestinian Territories is avoidable. Raising health awareness, gender equity, better outcomes of cataract surgery and improving accessibility should be targeted. The implementation of strategic and sustainable interventions in the delivery of eye services must be made a high priority.
